# Knowledge and personal values on fertility preservation procedures in Korean women

**DOI:** 10.1371/journal.pone.0331974

**Published:** 2025-10-24

**Authors:** Eun Mi Lee, Jieun Jang, Jaekyung Bae, Uisuk Kim, Sokbom Kang

**Affiliations:** 1 Division of Clinical Research, Research Institute, National Cancer Center, Goyang, Gyeonggi-do, Republic of Korea; 2 Center of Gynecologic Cancer, National Cancer Center, Goyang, Gyeonggi-do, Republic of Korea; 3 Center of Uterine Cancer, National Cancer Center, Goyang, Gyeonggi-do, Republic of Korea; King Saud University / Zagazig University, EGYPT

## Abstract

This study aimed to identify the knowledge of young general Korean female population on reproductive aging, as well as to investigate the personal values regarding fertility preservation (FP). A cross-sectional online survey study of 500 Korean female population on reproductive aging (18–40 years old) was performed. Knowledge regarding fertility preservation and its procedures was generally low. Correct knowledge about FP procedures was less than 50% for most of the questions. The rate of incorrect answers regarding FP knowledge overpassed 50%, which were related to loss of fertility and birth defects due to cancer treatment, and the rate of successful pregnancy for egg and embryo freezing. We also identified the personal values that influenced the willingness of women to undergo FP procedures. These included the following: 1) pregnancy success rate; 2) side effects of FP procedures; 3) delay of cancer treatment due to FP procedure; 4) risk of cancer recurrence; and 5) medical costs of FP procedures. Over 80% of the participants considered all five values important, with almost no distinctions. Despite the general awareness of FP, this study highlights the lack of knowledge and significant misconceptions regarding FP relative to fertility and cancer treatment among Korean women, which should be addressed properly with targeted education. Additionally, this study demonstrates diverse personal values participants hold regarding FP and the desire their values to be respected during the decision-making process of FP procedure facilitating shared decision-making (SDM).

## Introduction

Cancer treatments, such as chemotherapy, radiation, and certain surgeries often leave women with diminished reproductive potential. Therefore, fertility preservation (FP) is a rapidly evolving field dedicated to address the reproductive needs of patients diagnosed with cancer or other medical conditions that require treatments known to risk future fertility. FP procedure options including embryo and oocyte cryopreservation and ovarian tissue freezing can be vital interventions for preserving fertility in such patients [1]. Despite the growing importance and availability of FP procedures in cancer patients, studies indicate that only a minority of eligible patients are referred to reproductive endocrinologists for FP counseling, which highlights the need for building an environment that facilitates active FP discussions in oncology care [2,3].

Many women expressed the strong desire to preserve their ability to have children even though cancer treatment could impact fertility [4,5]. It is crucial that oncologists provide education and counseling regarding potential effects of cancer treatments on fertility, as well as the available FP procedure options including risks, benefits, and potential side effects. A recent study on fertility counseling revealed that such counseling improved patients' quality of life and psychological well-being in terms of reducing fertility-related distress [6].

However, although fertility issues are important concerns for female cancer patients during treatment, recent studies have shown that cancer patients possess limited knowledge about FP procedures. In a study of 410 breast cancer patients, 44.1% have heard of FP procedures, but only 9.1% were aware of the FP procedure options [7]. In another study of 70 breast cancer patients, there were patients who did not consider FP procedures due to insufficient provided information (30%) and incorrect knowledge regarding FP procedures, which included: beliefs FP procedure increased risk of cancer recurrence (46.0%) and unavailability of FP procedures (38.6%) [8]. This lack of knowledge often led women to miss opportunities to undergo FP procedures, because they were not informed in a timely manner about their options [7,8]. Many women expressed their uncertainty about the effectiveness and feasibility of FP procedures, which contributed to delays in their decision-making when considering these options [9].

Furthermore, the general population copes with inaccurate medical information by primarily relying on their healthcare professionals, who do not take into account the needs and concerns of patients referred to as personal values during the decision-making process [10,11]. To support SDM, it is essential to ensure population's current knowledge, identify gaps, ensure availability of accurate information, and understand the personal values women prioritize in medical decision-making [12].

Our study aimed to assess the level of knowledge regarding FP procedures among Korean women of reproductive age and explore the importance of personal values in the decision-making process regarding FP procedure. Comprehending the personal values that influence women's decisions such as pregnancy success rates or the possible side effects of FP procedures are crucial for developing personalized counseling strategies. This approach would help healthcare professionals address individual concerns, clarify misconceptions, and improve FP related services.

## Methods

### Study design and participants

The study was a cross-sectional online survey conducted from June 3^rd^ 2024 to June 28^th^ 2024, employed to Korean women aged between 18 and 40 years. Speculating that the proportion of women willing to undergo FP procedure is 50%, the survey sample size that can represent the population is 385 participants [13]. Supposing that approximately 20% of the responses are insufficient or incomplete, the determined sample size was 500. Participants were recruited via an online survey link sent to female members of a panel from an online survey company. After women younger than 18 years and older than 40 years were excluded, all participants provided written informed consent before enrollment and were asked to complete a questionnaire consisting of three sections: sociodemographic factors, knowledge, and personal values related when pursuing FP procedure. The study was approved by the Institutional Review Boards (IRB) of the National Cancer Center Korea (IRB approval number 2024-0202-0001).

### Outcome measures

#### Sociodemographic and personal information.

The survey collected sociodemographic information including age, province of residence, relationship status, level of education, monthly household income, and religious affiliation. The median monthly household income in Korea is approximately 5.000.000 won, which rounds towards 3400 USD approximately [14]. Additionally, participants were asked about their experience with childbearing, familiarity with the terms “fertility preservation” and “oocyte/embryo cryopreservation,” prior experience with FP procedures, and their intentions regarding future childbearing or pursuing a FP procedure.

#### Knowledge.

The knowledge section of the questionnaire was designed based on a review of existing literature to identify common knowledge areas related to FP procedures [7,15–17]. Subsequently, medical professionals, including 12 gynecologists and oncologists, were consulted to ensure the relevance and validity of the selected items. A two-round Delphi process was conducted to finalize the inclusion of 12 items, which assessed the knowledge of FP procedures. The items' key topics were risk of infertility due to cancer treatment, potential for birth defects associated with anticancer drugs, eligibility criteria for FP procedure, timing and process of FP procedure, pregnancy success rates, and use of FP procedure in children and adolescents. Participants were asked to respond to each item with a “yes,” “no,” or “not sure.” Participants selected “not sure” if they did not feel confident answering the question or believed they lacked the necessary knowledge.

#### Personal values.

The selection of personal value items was informed by two steps including a narrative interview on cancer patients who underwent FP counseling and a review of previous studies that explored factors that influenced patients' decisions undergoing FP procedures [18–21]. In one-on-one semi-structured interview, ten cancer patients of reproductive age participated to discuss the personal values they considered most important when deciding to undergo FP procedures. The most frequently mentioned values included pregnancy success rates, side effects associated with FP procedures, potential delays in cancer treatment due to FP procedure, risk of cancer recurrence, and medical costs associated with FP procedures.

Using the data full saturation method, we listed the personal values identified in the narrative interviews and literature review, and determined the most relevant values to include in the questionnaire through a two-round Delphi process with fertility preservation experts. The selected five personal values were: 1) side effects of FP procedures; 2) risk of cancer recurrence; 3) pregnancy success rate; 4) delay of cancer treatment due to FP procedures; and 5) medical costs associated with FP procedures. A five-item questionnaire was developed to assess the importance of these values, using a 5-point Likert scale: “not important at all,” “not important,” “neutral (undecided),” “important,” and “very important.”

## Results and discussion

### Sociodemographic characteristics

After all participants who did not meet the inclusion criteria were excluded, a total of 500 women aged between 18 and 40 years participated in the online survey (Table 1). Most participants (98.8%) had attained at least a high school education, and more than half (51.8%) reported a household monthly income above the median for South Korea. Most participants had not experienced childbearing (71.2%). Regarding familiarity with the term “fertility preservation,” 65.8% of the participants never have heard of the term. In contrast, 63.6% of the participants were familiar with the terms “oocyte/embryo cryopreservation,” understood its meaning, and felt confident and able to explain the terms to others. The majority of participants (97.6%) had never received FP procedure, and 43.0% expressed a desire for childbearing but indicated no interest in pursuing FP procedure.

**Table 1:** Participants’ sociodemographic characteristics and personal information on fertility preservation (N = 500).

Variable	Categories	N	%
Age	18-19 years	19	3.8
	20-29 years	222	44.4
	30-40 years	259	51.8
Area of residence (province)	Seoul	116	23.2
	Busan	18	3.6
	Daegu	30	6.0
	Incheon	29	5.8
	Gwang-ju	18	3.6
	Daejeon	12	2.4
	Ulsan	12	2.4
	Gyeonggi-do	142	28.4
	Gangwon-do	13	2.6
	Chungcheongbuk-do	13	2.6
	Chungcheongnam-do	20	4.0
	Jeollabuk-do	14	2.8
	Jeollanam-do	12	2.4
	Gyeongsangbuk-do	10	2.0
	Gyeongsangnam-do	36	7.2
	Jeju-do	4	0.8
	Sejong	1	0.2
Relationship status	Single	238	47.6
	Married/Partnered	250	50.0
	Divorced/Widowed	12	2.4
Level of education	No education	0	0.0
	Elementary school	0	0.0
	Middle school	6	1.2
	High school or above (undergraduate/graduate school)	494	98.8
Household monthly income	Less than 5.000.000 won (3.400 US dollars approximately)	241	48.2
	More than 5.000.000 won (3.400 US dollars approximately)	259	51.8
Experience of childbearing	Yes	144	28.8
	No	356	71.2
Familiarity with the term "fertility preservation"	Never heard of the term fertility preservation	329	65.8
	Aware of the term "fertility preservation" but unaware of its meaning	100	20.0
	Aware of the term "fertility preservation" and its meaning, but unable to explain to others	66	13.2
	Aware of the term "fertility preservation", its meaning, and able to explain to others	5	1.0
Familiarity with the term "oocyte/embryo cryopreservation"	Never heard of the term "oocyte/embryo cryopreservation"	35	7.0
	Aware of the term "oocyte/embryo cryopreservation" but unaware of its meaning	83	16.6
	Aware of the term "oocyte/embryo cryopreservation" and its meaning, but unable to explain to others	318	63.6
	Aware of the term "oocyte/embryo cryopreservation", its meaning, and able to explain to others	64	12.8
Have received fertility preservation	Never have received fertility preservation	488	97.6
	Have received oocyte cryopreservation	5	1.0
	Have received ovary cryopreservation	1	0.2
	Have received ovarian tissue cryopreservation and transplant surgery	1	0.2
	Have received ovary repositioning surgery	2	0.4
	Have administered gonadotropin-releasing hormone (GnRH) hormone inhibitors	0	0.0
	Unknown/Unable to remember	3	0.6
Desire of childbearing or receiving fertility preservation	No interest in childbearing or receiving fertility preservation	184	36.80
	No interest in childbearing, but desire of receiving fertility preservation	36	7.2
	Desire of childbearing, but no interest in receiving fertility preservation	215	43.0
	Desire of childbearing and receiving fertility preservation	65	13.0
Religion	Christianity	78	15.6
	Catholicism	31	6.2
	Buddhism	40	8.0
	Other/Atheism	351	70.2

### Knowledge of FP procedures

The participants' knowledge of FP was generally low (Table 2). Among the twelve questions related to the risk of fertility loss due to cancer treatment, conditions and process for FP procedures, success rates of FP procedures, and FP procedures in pediatric and adolescent cancer patients, only two questions about the impact of FP procedures on successful pregnancy rates had a correct response rate of over 70%. Participants were aware that embryo freezing and egg freezing FP procedures does not guarantee pregnancy. For most of other questions, less than 50% of the participants provided correct answers. Notably, more than 50% of the respondents incorrectly answered questions related to the negative effects of cancer treatments on fertility and the comparison of success rates between embryo freezing and egg freezing. Specifically, 50.4% of the participants believed that all cancer treatments caused fertility loss or fetal disability. Moreover, 52.2% of the participants incorrectly assumed that the probability of successful pregnancy differs between embryo freezing and egg freezing.

**Table 2:** Participants’ knowledge regarding fertility preservation (*N* = 500).

Question	Correct	Incorrect	Not Sure
	*N* (%)		
All cancer treatments can reduce a cancer patient's chances of becoming pregnant or cause infertility	70 (14.0)	252 (50.4)	178 (35.6)
Most anticancer drugs are known to cause birth defects	62 (12.4)	266 (53.2)	172 (34.4)
Cancer patients who are not married or do not have a spouse can also choose fertility preservation procedure	285 (57.0)	57 (11.4)	158 (31.6)
All fertility preservation procedures should be performed before starting cancer treatment	77 (15.4)	222 (44.4)	201 (40.2)
When a cancer patient freezes embryos to preserve fertility, she will attempt to get pregnant through in vitro fertilization	231 (46.2)	60 (12.0)	209 (41.8)
Women who have frozen their eggs can use them anytime within the storage period	315 (63.0)	69 (13.8)	116 (23.2)
The success rate of pregnancy is the same for all types of fertility preservation procedures	296 (59.2)	28 (5.6)	176 (35.2)
All women who freeze their eggs or embryos can successfully become pregnant	371 (74.2)	16 (3.2)	113 (22.6)
Egg freezing and embryo freezing have the same probability of a successful pregnancy	30 (6.0)	261 (52.2)	209 (41.8)
Receiving a fertility preservation procedure does not necessarily mean that future pregnancies will be successful	392 (78.4)	37 (7.4)	71 (14.2)
There are fertility preservation procedures for children and adolescent cancer patients who have not yet had menarche	36 (7.2)	154 (30.8)	310 (62.0)
To freeze eggs, you must have your spouse's consent	217 (43.4)	112 (22.4)	171 (34.2)

Additionally, a significant proportion of participants (44.4%) incorrectly believed that all FP procedures must be performed before starting cancer treatment. When they were asked whether FP procedures could be performed for pediatric and adolescent cancer patients before experiencing menarche, 62% of the respondents answered, “not sure.” This finding highlights the fact that FP procedures for younger cancer patents remain as a largely unfamiliar concept among the general population.

### Personal values regarding FP procedures

This study also explored the personal values that Korean women prioritize when considering FP procedures (Table 3). The five personal values identified were as follows: 1) side effects of FP procedures; 2) risk of cancer recurrence; 3) pregnancy success rate; 4) delay of cancer treatment due to FP procedures; and 5) medical costs associated with FP procedures. Over 80% of participants rated all five values as being significantly important, with minimum dissimilarity.

**Table 3:** Personal values among participants regarding FP procedures (*N* = 500).

Variable	Question	Not important at all	Not important	Neutral	Important	Very important
		n (%)				
*Side* *effects*	The types and levels of side effects for each fertility preservation procedures are different. How important are the **side effects** you may experience from fertility preservation procedure affect your decision?	0 (0.0)	3 (0.6)	57 (11.4)	185 (37.0)	255 (51.0)
*Risk of cancer* *recurrence*	Some fertility preservation procedures may increase the chance of cancer recurrence. How much does the probability of **cancer recurrence** due to fertility preservation procedure affect your decision?	1 (0.2)	7 (1.4)	53 (10.6)	167 (33.4)	272 (54.4)
*Pregnancy success rate*	The success rate of pregnancy varies depending on the type of fertility preservation procedure. How important is the **pregnancy success rate** when choosing your fertility preservation procedure?	6 (1.2)	6 (1.2)	60 (12.0)	259 (51.8)	169 (33.8)
*Delay in* *treatment*	Some fertility preservation procedures may require delay in cancer treatment. How important is the **delay in cancer treatment** in your decision?	1 (0.2)	9 (1.8)	75 (15.0)	219 (43.8)	196 (39.2)
*Cost of FP* *procedure*	Medical costs vary depending on the type of fertility preservation procedure. How important is the **cost** for undergoing fertility preservation in your decision?	4 (0.8)	12 (2.4)	83 (16.6)	204 (40.8)	197 (39.4)

Although there was no significant difference in importance between the five identified values, minimum distinction was revealed (Table 3). The participants indicated the importance of personal values they considered when deciding to receive FP procedure as follows: 1) side effects of FP procedures (88.0%); 2) risk of cancer recurrence (87.8%); 3) pregnancy success rate (85.6%), 4) delay of cancer treatment due to FP procedures (83.0%), and 5) medical costs associated with FP procedures (80.2%).

## Discussion

The participants in the study were generally familiar with the concept of FP procedures, but many were unable to accurately explain its meaning or describe the procedures involved. This finding points to a broader issue of limited awareness and understanding of medical procedures even among individuals who have heard of the concept, which might plausibly lead to poor decision-making on the basis of incomplete or inaccurate information.

Similar findings have been reported in a recent study. While many women (44.1%) were aware of FP procedure options, few (9.1%) had a clear understanding of what these options involved [7]. The exposed information about FP procedures, most were from sources such as the internet, media, and peer networks instead of acquiring the information through physicians and scientific papers [8]. This emphasizes the need for improved communication strategies and a more active role from healthcare professionals in educating patients about the available options and potential consequences of delaying or foregoing FP procedures.

The lack of knowledge among the participants in our study is concerning, especially given the growing importance of FP procedures in various contexts. This problem is especially relevant for women with cancer or other illnesses that might affect their fertility. While cancer treatments drastically impact fertility, FP procedures give women the opportunity to preserve their fertility [22]. Therefore, providing clear and accurate information about FP procedures is essential to facilitate SDM, especially for women with medical problems or those facing challenges in making health-related decisions.

Another finding in the study highlights the importance of personal values in women's decision-making process regarding FP procedures. Respectively, concerns of potential side effects and the risk of cancer recurrence were identified as the most frequent and influential factors in their decision-making. This aligns with previous research on decision-making in medical contexts, where side effects, safety, and long-term health outcomes often outweigh other factors such as medical costs or pregnancy success rates [23].

Our study highlights the primary concern of Korean women seemed to be related to medical risks that impact their health and well-being. This finding emphasizes the importance of educating patients from a more interactive and empathetic perspective, encouraging open communication that meets patients' needs and preferences [24]. This approach may help and guide women make better decisions of FP procedures or other medical situations that may affect their fertility.

In our study, surveying the general population aided in identifying their information needs for FP and SDM. Surveying the general population may facilitate the capture of baseline knowledge and perceptions regarding FP before cancer diagnosis. Pre-diagnostic awareness allows for the development of educational tools and helps prepare individuals before reaching to urgent decision-making and enhance SDM [23,24]. In addition, surveying the general population provides diverse values and preferences that women possess regarding FP [23].

However, relying solely on the data from general population may overlook the concerns of cancer patients, such as survival, recurrence risks, and treatment efficacy. The emotional burden and psychological stress faced by cancer patients can significantly influence their information needs and decision-making process, which makes it challenging to survey only the general population [6,24]. Nevertheless, the general population provides valuable baseline insights, but healthcare professionals should ensure decision aids that address the specific needs of cancer patients.

Due to the design of this study, there were few limitations. Despite the efforts to ensure representativeness of the study results by sampling subjects based on the population structure in Korea, there may still be selection bias because the subjects were recruited from a panel of an online survey company. In addition, the percentage of women with a high school education or higher (98.8%) in the study was higher than the average level of education in Korea (45.4% in 2022) [25]. Therefore, the participants in this study had higher education level, which may have resulted in an overestimation of FP knowledge posing a challenge to establish representativeness.

## Conclusions

Korean women were generally aware of the FP procedures, but significant gaps between awareness and real knowledge persisted. The understanding of FP procedures and their impact on reproductive health remained unclear, with visible misconceptions regarding FP procedures due to insufficient communication and education. Moreover, concerns about side effects and risk of cancer recurrence associated with FP procedures, significantly influenced in the decision-making. To address these gaps, educational interventions and a more proactive role of healthcare professionals during patient counseling are needed.

In addition, it is vital that FP procedures counseling provide medical information, emotional support, and ensured choices. Most importantly, identifying and accounting patients' personal values should be guaranteed during FP counseling to proceed with SDM. By integrating patients' personal values, healthcare professionals can deliver more individualized, culturally sensitive, and respectful care leading to better patient outcomes and satisfaction. This study underscores the urge of an integrated approach to patient education in medical, emotional, and psychological aspects, to support women in making a more effective SDM regarding FP procedures.

## Supporting information

S1 File This is the S1 File Raw dataset. (XLSX)

## List of abbreviations:

FP: fertility preservation
SDM: shared decision-making
